# Identification of the Antibacterial Compound Produced by the Marine Epiphytic Bacterium *Pseudovibrio* sp. D323 and Related Sponge-Associated Bacteria

**DOI:** 10.3390/md9081391

**Published:** 2011-08-12

**Authors:** Anahit Penesyan, Jan Tebben, Matthew Lee, Torsten Thomas, Staffan Kjelleberg, Tilmann Harder, Suhelen Egan

**Affiliations:** 1 School of Biotechnology and Biomolecular Sciences and Centre for Marine Bio-Innovation, University of New South Wales, Sydney 2052, New South Wales, Australia; E-Mails: anahit.penesyan@mq.edu.au (A.P.); mattlee@unsw.edu.au (M.L.); t.thomas@unsw.edu.au (T.T.); s.kjelleberg@unsw.edu.au (S.K.); 2 School of Biological, Earth and Environmental Sciences and Centre for Marine Bio-Innovation, University of New South Wales, Sydney 2052, New South Wales, Australia; E-Mails: jan.tebben@student.unsw.edu.au (J.T.); t.harder@unsw.edu.au (T.H.); 3 Singapore Centre on Environmental Life Sciences Engineering, Nanyang Technological University, 60 Nanyang Drive, SBS-01n-42, 637551, Singapore

**Keywords:** marine bacteria, bioactive, antimicrobial, symbiosis, tropodithietic acid

## Abstract

Surface-associated marine bacteria often produce secondary metabolites with antagonistic activities. In this study, tropodithietic acid (TDA) was identified to be responsible for the antibacterial activity of the marine epiphytic bacterium *Pseudovibrio* sp. D323 and related strains. Phenol was also produced by these bacteria but was not directly related to the antibacterial activity. TDA was shown to effectively inhibit a range of marine bacteria from various phylogenetic groups. However TDA-producers themselves were resistant and are likely to possess resistance mechanism preventing autoinhibition. We propose that TDA in isolate D323 and related eukaryote-associated bacteria plays a role in defending the host organism against unwanted microbial colonisation and, possibly, bacterial pathogens.

## 1. Introduction

Ever since the discovery of penicillin there have been many attempts to find novel antimicrobials due to the inevitable development of bacterial resistance towards the widely used antibiotics. In the past the vast majority of natural product research focused on terrestrial sources. Nowadays, the exploration of new and under-explored sources becomes extremely important in the process of finding biologically active compounds (“bioactives”) that can be used as new antibiotics [1]. In recent years microorganisms associated with the surfaces of marine eukaryotes have been major targets for the discovery of new bioactive metabolites [2,3]. This is primarily due to the notion that both microorganisms and hosts have developed a range of chemical defence strategies for the fiercely competitive environment in which they reside [2–4]. One such microbe that has pronounced antibacterial activity is the bacterial strain D323, recently isolated from the temperate red alga *Delisea pulchra* [5,6].

The present study aimed to (1) establish the phylogenetic diversity of isolate D323 and closely related bacteria; (2) identify the causative compound responsible for the antibacterial activity; and (3) assess the spectrum of activity of the antibacterial compound in relation to the possible ecological role for its production.

## 2. Results and Discussion

### 2.1. Isolate D323 Is a Representative of a Widespread, Surface-Associated *Alphaproteobacteria* Clade

**Figure 1.**
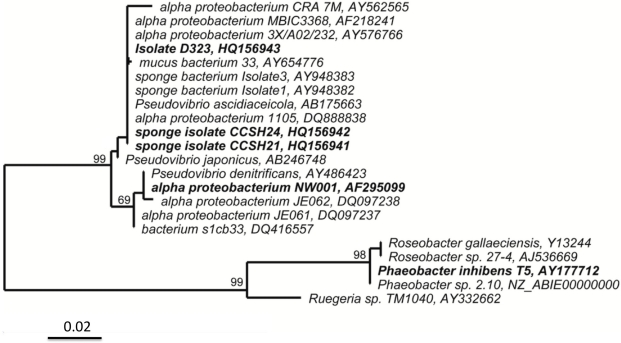
Maximum likelihood phylogenetic tree based on 16S rRNA gene sequence showing the isolates used in this study (in bold) and their close neighbours from GenBank/EMBL/DDB related to isolate D323. Maximum parsimony bootstrap values (1000 resamplings) are given for major nodes. The scale bar indicates the number of substitutions per nucleotide position.

Phylogenetic analysis of 16S rRNA gene sequence of isolate D323 revealed that it is an *Alphaproteobacterium* belonging to the family *Rhodobacteraceae*, with the species *Pseudovibrio ascidiaceicola* being the closest fully described relative (99% identity with the type strain, Genbank/EMBL/DDBJ Acc. #AB175663) [7] (Figure 1). In contrast to *P. ascidiaceicola*, which grows as smooth brown-green colonies, isolate D323 form flat brown mucoid colonies when grown on Marine Agar (Difco 2216) and resembles that of isolate MBIC3368 and related sponge-associated bacteria, to which it has 98% identity in the 16S rRNA gene sequence [8–10] (Figure 1). Antimicrobial production was reported for another *Pseudovibrio* related to *P. denitrificans*, strain Z143-1; the compound was identified as heptylprodigiosin [11]. Moreover, the colony morphology of that strain, which was reported to form red colonies while producing the bioactive compound, differed significantly from the above-mentioned colony morphology of isolate D323 and MBIC3368 related bacteria [8–10]. Antibacterial compounds are likely to be produced by yet other *Pseudovibrio* species as Geng and Belas (2010) recently identified genes encoding for the production of the antibacterial compound tropodithietic acid (TDA) in strain *Pseudovibrio* sp. strain JEO62, which forms a distinct clade from D323 [12] (Figure 1).

Bacterial isolates related to *Pseudovibrio* sp. D323 have previously been reported to be associated with different marine sponges in various geographical regions [10,13,14] and not detected in the surrounding seawater [9], suggesting possible host specificity. Moreover, it has been suggested that these bacteria play an important role in host defence against disease as they were found to be present in healthy sponges but absent in diseased individuals [9,15]. In addition, Enticknap *et al.* [9] demonstrated that the D323-related bacterium NW001 could be vertically transferred from the parent sponge to the offspring, further emphasizing its importance for the host. Despite reports showing antibacterial activity in some D323-related isolates [10,16], earlier attempts to chemically identify the bioactive compound/s produced by these bacteria were not successful [10].

### 2.2. Identification of Antibacterial Secondary Metabolites in D323 and Related Strains

**Figure 2.**
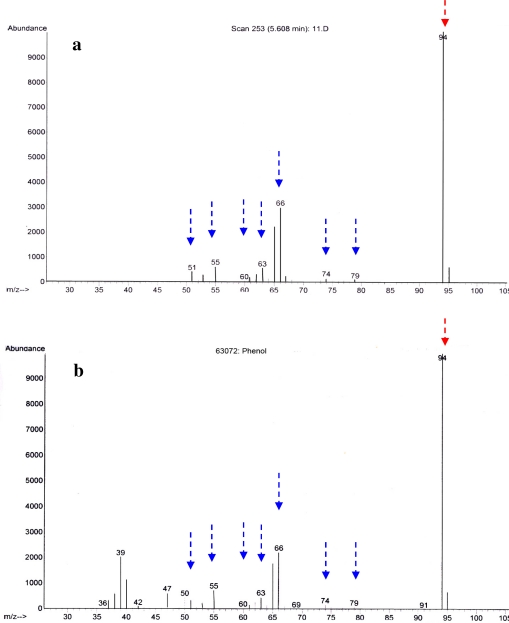
GC-MS analysis of isolate D323 crude extract (**a**) and corresponding MS spectrum of phenol (**b**) Identical masses are indicated by dashed arrows, the mass of the parental molecule (phenol) is indicated by the red arrow. Data was collected from *m/z* 45 to 105.

**Figure 3.**
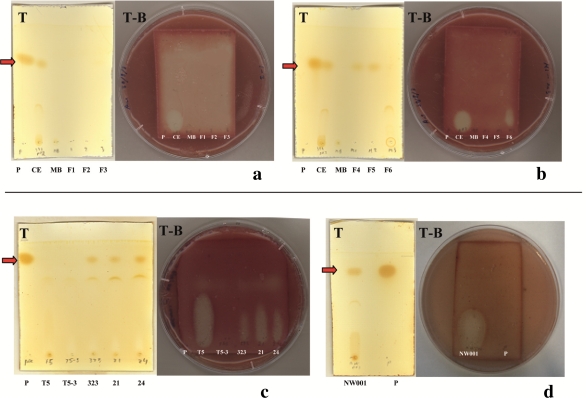
Results of TLC (T), TLC-BOA (T-B) of crude ethylacetate extract (CE), of isolate D323 (323), CCSH21 (21); CCSH24 (24); NW001; *P. inhibens* T5 (T5) and its TDA-deficient mutant *P. inhibens* T5-3 (T5-3) grown in MB (**a**–**d**); as well as of the fractions F1–F6 obtained via the fractionation of the crude extract of isolate D323 (**a**, **b**). The ethylacetate extract of acidified MB medium (MB) and phenol (P, 0.1 mg/mL in ethylacetate, Sigma) were used as controls. The red arrow indicates the position of phenol on the TLC plate after developing it in ethylacetate:hexane (5:1) mixture.

Given the widespread occurrence of the D323-related sequences and their suggested importance for the host, as well as the potential for new drug discovery, the identification of its antibacterial activity was of great interest. The antibacterial activity was found in the spent medium of the liquid culture of *Pseudovibrio* sp. D323. Analysis of the *Pseudovibrio* sp. D323 crude extract by GC-MS identified phenol as a major component by both specific fragmentation pattern and a GC retention time (5.608 min) for the alleged phenol peak in the crude extract which matched that of standard made with phenol (Figure 2). However, as shown by TLC and TLC-BOA, the position of the halo, which corresponded to the position of antibacterial compound, was different from the position of phenol (Figure 3a,b) suggesting that the antibacterial activity, observed in *Pseudovibrio* sp. D323 crude extract, was not due to phenol.

**Figure 4.**
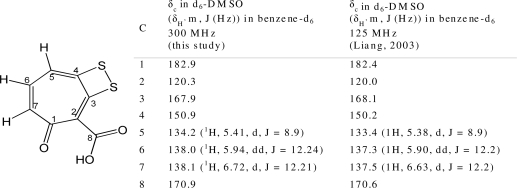
Molecular structure of **1** (tropodithietic acid) and NMR spectroscopic data (ppm) of **1** in comparison to published data by Liang, 2003 [17]. ^13^C experiments were obtained in d_6_-DMSO, ^1^H experiments were obtained in benzene-d_6_ on a Bruker Avance III 300 MHz.

Further purification and subsequent chemical analysis of the active fraction using LC-MS and NMR identified the presence of tropodithietic acid (TDA, up to 1.6 mg/L of spent medium) [17,18] (Figure 4). TDA is a known antimicrobial compound produced by *Phaeobacter inhibens* and related strains within the *Roseobacter* clade [18–21]. Our data correlates with the recent prediction of TDA production in other *Pseudovibrio* strains based on the presence of homologs to the biosynthetic genes (*tdaA–tdaF*), which were also found to be positively regulated in the presence of TDA producers [12].

To confirm that D323-related strains indeed produce TDA, crude extracts of sponge-associated isolates, CCSH21, CCSH24, NW001, as well as a known TDA producer, *P. inhibens* were assessed via TLC. The TLC profile of CCSH21, CCSH24 and NW001 were identical to *Pseudovibrio* sp. D323 (Figure 3c,d), also showing the presence of phenol. In contrast to *Pseudovibrio* sp. D323, no phenol was detected either in *P. inhibens* or the TDA-deficient mutant *P. inhibens* T5-3 grown in the same medium (Figure 3c,d). This data correlates with a recent report showing the absence of phenol in *P. inhibens* cultures grown in MB medium, while only trace amounts of phenol were observed in the medium supplemented with phenylacetic acid [22]. The production of TDA was evident by the zone of inhibition in TLC-BOA, in all the crude extracts of isolates grown in MB, with the exception of *P. inhibens* T5-3 (Figure 3). Phenol production was observed in all the isolates related to *Pseudovibrio* sp. D323 and was not detected in *P. inhibens* (Figure 3c,d). The presence of phenol and/or TDA was confirmed by HPLC (data not shown).

Interestingly, phenol production has been mainly reported in enteric bacteria [23]. Synthesis of phenol by those bacteria is carried out by the enzyme tyrosine-phenol-lyase (TPL) [24–26] and is directly linked to the availability of l-tyrosine. To assess whether the production of phenol by *Pseudovibrio* sp. D323 correlated with the presence of l-tyrosine, TLC analysis of the spent medium of the strain grown in the presence and absence of l-tyrosine was performed. Trace amounts of phenol were observed in the crude ethylacetate extract of *Pseudovibrio* sp. D323 grown in MMM, whereas phenol production was greatly enhanced in the presence of l-tyrosine (data not shown). This is suggestive of a similar pathway for phenol biosynthesis in *Pseudovibrio* sp. D323 as has been reported for enteric bacteria.

The precise role of phenol production and secretion by *Pseudovibrio* sp. D323 and related bacteria is not clear. Despite being a known disinfectant, phenol, even at concentration of 1 mg/mL, did not have antibacterial activity in our assays. It is possible, however, that phenol may be used by the host as a precursor for the synthesis of yet other phenolic-based defence compounds common in many marine sponges and algae [27–31].

### 2.3. TDA Inhibits a Variety of Marine Bacteria but Shows No Auto-Inhibition in Producer Strains

In order to assess the inhibitory spectrum of TDA against a variety of relevant environmental strains, disc diffusion assays [32], using a concentration of 0.1 mg/mL TDA, were performed against a collection of marine strains, including various *D. pulchra* associated bacteria. TDA inhibited a wide range of strains, including isolates belonging to all major phyla found on marine living surfaces, such as the *Proteobacteria*, *Actinobacteria*, *Firmicutes* and *Bacteroidetes* (Table 1). These results differ from an earlier study [19], which observed a phylogenetic bias with respect to the sensitivity to TDA of isolates from the German Wadden sea. Specifically, in that study *Gammaproteobactera*, were found to be mostly resistant to TDA [19], while our results show that bacteria from this class were among the most sensitive. To date nothing is known about the mode of action of TDA and it is, therefore, possible that differences of the target isolate at the level of genera and species may cause the observed differences.

It is interesting that the only strains found to be resistant to the inhibitory effect of TDA are TDA-producers themselves, including strain D323, *P. inhibens*, and the *P. gallaeciensis* strains 2.10 and BS107 [19]. Bruhn *et al.* [33] also observed *Roseobacter* clade strains to be TDA-resistant whilst all non-*Roseobacter* strains tested were sensitive to TDA produced by *Ruegeria* sp. (formerly *Silicibacter*) TM1040 [34]. Together these observations suggest that TDA producers may possess an efficient resistance mechanism to prevent auto-inhibition. Notably, the TDA-deficient mutant of *P. inhibens*, T5-3, still maintains this resistance characteristic (Table 1).

The wide activity spectrum of activity of TDA suggests that its production can serve as a defence against competitive bacteria, and, hence, could protect the host against other surface colonisers. Notably, TDA was shown to be highly active against *Nautella* sp. R11 (formally *Ruegeria* sp. R11) (Table 1), which causes a bleaching disease in *D. pulchra* [35]. The observation of Webster *et al.* [15] that the loss of the TDA-producer NW001 coincides with heavy microbial colonisation of the sponge, subsequently leading to its death, may also support this proposition. Whether TDA is produced in sufficient quantities in the natural environment to be able to protect the eukaryotic host as well as producer microorganisms from other potentially pathogenic colonizers remains to be a subject for a future study.

## 3. Experimental Section

### 3.1. Bacterial Strains and Culture Conditions

Isolate D323 was obtained from the surface of the red alga *Delisea pulchra* [6]. Isolates CCSH21 and CCSH24 are from the surface of the temperate sponge *Cymbastela concentrica* [36] located in coastal waters near Sydney, Australia. The *Alphaproteobacterium* NW001 was isolated from the tropical sponge *Rhopaloeides odorabile* [8] from the Great Barrier Reef. *Phaeobacter inhibens* T5 was isolated from marine sediment in Wadden Sea, Germany and *P. inhibens* T5-3 is a TDA deficient spontaneous mutant of T5 [19]. All strains were grown routinely in Marine Broth (MB, Difco 2216) shaking at ambient room temperature.

### 3.2. 16S rRNA Gene Sequencing and Analysis

To determine the phylogenetic relationship among D323-related strains, the near-full-length 16S rRNA gene sequence of D323 was obtained using standard procedures [6] with the following universal primers: F27 (5′-GAGTTTGATCCTGGCTCAG-3′) [37], R1492 (5′-ACGGTTACCTTGTTACGACTT-3′) [38], F530 (5′-GTGCCATC-CAGCCGCGG-3′) and R903 (5′-CCGTCAATTCCTTTRAGTTT-3′) [39]. The consensus 16S rRNA gene sequence was used for the phylogenetic comparisons and a maximum likelihood tree was constructed using the ARB software [40].

### 3.3. Antibacterial Assays and Preparation of Crude Antibacterial Extracts

Antibacterial assays were performed using a disc diffusion method [32] with *Neisseria canis* OH73 as a target strain due to its high sensitivity to strain D323 [6].

To prepare initial antibacterial crude extracts from strain D323 and related bacterial strains, cultures were grown in MB to stationary phase. Spent medium was then collected and acidified to pH 2 using hydrochloric acid followed by extraction with ethylacetate. To assess the impact of l-tyrosine on the production of phenol, strain D323 was grown in marine minimal medium (MMM) [41] with and without the addition of l-tyrosine (0.9 mM) prior to extraction of the spent medium as described above.

### 3.4. Gas-Chromatography Mass-Spectrometry (GC-MS)

The antibacterial crude extract was analysed by GC-MS using a 5890 series II gas chromatograph (Hewlett Packard), with a DB5 30 m × 0.32 mm (internal diameter) × 250 mm (film thickness) column, coupled to a 5972A mass-spectrometer (Hewlett Packard). The inlet temperature was set at 250 °C; with a split ratio of 50:1. The GC oven was operated at an initial temperature of 50 °C for 1 min and then increased to 200 °C at a rate of 20 °C/min. The transfer line temperature was set to 250 °C. Data were collected from *m/z* 45 to 105.

### 3.5. Thin Layer Chromatography (TLC) and TLC-Bioautography Overlay Assay (TLC-BOA)

Crude extracts were spotted on TLC plates (Merck, silica gel 60 F254) in duplicate, along with relevant standards and media controls. The TLC plates were developed in ethylacetate:hexane (5:1) mixture, air-dried and stained in iodine vapour for 5 min to visualise sample spots. The duplicate plates were used for the TLC-BOA [42], in which after the development the TLC plate was overlayed with soft Luria Bertani (LB) agar containing 0.01% (w/v), 2,3,5-triphenyl-tetrazolium chloride (TTC, Sigma), and inoculated with a 5% (v/v) overnight culture of the target strain *N. canis* OH73. The overlayed plate was incubated at 37 °C overnight. Antibacterial activity was observed as a transparent halo on a red stained background that was indicative of bacterial growth.

### 3.6. Purification of the Antibacterial Compound

Initial purification of the active antibacterial compound was performed via solid phase extraction using prepacked cartridges (Silica, 10 g, 60 mL, Alltech). Five fractions (F1–F5) were eluted with an ethylacetate:hexane mixture, increasing the ratio of ethylacetate from 5:1 to a 10:1. The final eluate fraction (F6) was obtained with 100% methanol.

Further purification and subsequent chemical analysis of the active fraction using LC-MS and NMR is described in the supplementary material.

## 4. Conclusions

This study has demonstrated both phenol and TDA production in *Pseudovibrio* sp. D323 and related strains, the latter being responsible for the antibacterial activity of these bacteria. The wide activity spectrum of TDA against various bacteria, including common marine epibionts, indicates that TDA could be used by *Pseudovibrio* sp. D323 and related organisms to defend themselves against other competitors. The frequent isolation of *Pseudovibrio* sp. D323 from marine sessile eukaryotic hosts also indicates a role for these bacteria in the defence of the eukaryotic host against heavy colonisation.

## Supplementary Material



## Figures and Tables

**Table 1.** Inhibitory activity of TDA (0.1 mg/mL in methanol) produced by isolate D323 against various marine bacteria as tested using the disc-diffusion method.

**Target Strains**	**Phylum**	**Origin**	**Inhibition by TDA**
*Alpha-proteobacterium* D323 ^	*Alphaproteobacteria*	seaweed *Delisea pulchra*	−
*Phaeobacter inhibens* T5 ^	*Alphaproteobacteria*	marine sediment	−
*Phaeobacter inhibens* T5-3	*Alphaproteobacteria*	marine sediment	−
*Nautella* sp. R11	*Alphaproteobacteria*	seaweed *Delisea pulchra*	+++
*Phaeobacter gallaeciensis* 2.10 ^	*Alphaproteobacteria*	seaweed *Ulva lactuca*	−
*Oceanicola granulosus*	*Alphaproteobacteria*	seawater	++
*Oceanicola batsensis*	*Alphaproteobacteria*	seawater	+++
*Roseovarius* sp. 2601	*Alphaproteobacteria*	seawater	++
*Oceanicaulis alexandrii*	*Alphaproteobacteria*	seawater	++
*Rhodobacterales bacterium*	*Alphaproteobacteria*	seawater	+++
*Phaeobacter gallaeciensis* BS107 ^	*Alphaproteobacteria*	scallop *Pecten maximus*	−
*Vibrio harveyi*	*Gammaproteobacteria*	seawater	+++
*Pseudoalteromonas tunicata*	*Gammaproteobacteria*	tunicate *Ciona intestinalis*	+++
*Pseudoalteromonas undina*	*Gammaproteobacteria*	seawater	++
*Pseudoalteromonas piscicida*	*Gammaproteobacteria*	dead fish	+
*Pseudoalteromonas citrea*	*Gammaproteobacteria*	seawater	++
*Pseudoalteromonas haloplanktis*	*Gammaproteobacteria*	oyster *Crassostrea gigas*	++
*Pseudoalteromonas ulvae*	*Gammaproteobacteria*	seaweed *Ulva lactuca*	++
*Pseudoalteromonas flavipulchra*	*Gammaproteobacteria*	seawater	+
*Acinetobacter* sp. ESS07	*Gammaproteobacteria*	seaweed *Delisea pulchra*	+++
*Marinomonas* sp. ND73	*Gammaproteobacteria*	seaweed *Delisea pulchra*	+++
*Shewanella* sp. ND51	*Gammaproteobacteria*	seaweed *Delisea pulchra*	++
*Thalassomonas* sp. ND29	*Gammaproteobacteria*	seaweed *Delisea pulchra*	+++
*Thalassomonas* sp. ND49	*Gammaproteobacteria*	seaweed *Delisea pulchra*	++
*Aestuariibacter* sp. ND16	*Gammaproteobacteria*	seaweed *Delisea pulchra*	++
*Vibrio* sp. ND23	*Gammaproteobacteria*	seaweed *Delisea pulchra*	++
*Dokdonia* sp. ESS16	*Bacteroidetes*	seaweed *Delisea pulchra*	++
*Aquimarina* sp. ND19	*Bacteroidetes*	seaweed *Delisea pulchra*	+
*Tenacibaculum* sp. ND71	*Bacteroidetes*	seaweed *Delisea pulchra*	+
*Bacillus* sp. D203	*Firmicutes*	seaweed *Delisea pulchra*	++
*Bacillus* sp. ESS03	*Firmicutes*	seaweed *Delisea pulchra*	+++
*Micrococcus* sp. ESS26	*Actinobacteria*	seaweed *Delisea pulchra*	++
*Agrococcus* sp. LSS27	*Actinobacteria*	seaweed *Delisea pulchra*	++

+ zone of clearance in disc diffusion up to 3 mm from the edge of the disc;

++ 3–5 mm;

+++ more than 5 mm;

− no inhibition observed;

^ TDA-producing bacteria.
