# Supplementary material for: Identification of the Antibacterial Compound Produced by the Marine Epiphytic Bacterium Pseudovibrio sp. D323 and Related Sponge-Associated Bacteria
Source: Mar Drugs. 2011 Aug 12;9(8):1391–402. doi: 10.3390/md9081391 (PMC3164381; doi:10.3390/md9081391)
Supplement: Supplementary file 1 [file marinedrugs-09-01391-s001.pdf]

Supplementary Material

Article

Identification of the Antibacterial Compound Produced by the Marine Epiphytic Bacterium *Pseudovibrio* sp. D323 and Related Sponge-Associated Bacteria

Anahit Penesyan [1,†], Jan Tebben [2], Matthew Lee [1], Torsten Thomas [1], Staffan Kjelleberg [1,3], Tilmann Harder [2] and Suhelen Egan [1,*]

[1] School of Biotechnology and Biomolecular Sciences and Centre for Marine Bio-Innovation, University of New South Wales, Sydney 2052, New South Wales, Australia; E-Mails: anahit.penesyan@mq.edu.au (A.P.); mattlee@unsw.edu.au (M.L.); t.thomas@unsw.edu.au (T.T.); s.kjelleberg@unsw.edu.au (S.K.)

[2] School of Biological, Earth and Environmental Sciences and Centre for Marine Bio-Innovation, University of New South Wales, Sydney 2052, New South Wales, Australia; E-Mails: jan.tebben@student.unsw.edu.au (J.T.); t.harder@unsw.edu.au (T.H.)

[3] Singapore Centre on Environmental Life Sciences Engineering, Nanyang Technological University, 60 Nanyang Drive, SBS-01n-42, 637551, Singapore

[†] Current address: Department of Chemistry and Biomolecular Sciences, Macquarie University, Sydney 2109, New South Wales, Australia.

* Author to whom correspondence should be addressed; E-Mail: s.egan@unsw.edu.au; Tel.: +61-2-9385-8569; Fax: +61-2-9385-1779.

Purification and Chemical Characterisation of TDA

To further purify the antibacterial compound, the spent culture medium of overnight liquid culture of *Pseudovibrio* sp. D323 was exhaustively extracted, concentrated under vacuum, yielding an orange-red residue. The residue was partitioned in acidified 60% aqueous methanol (0.05% trifluoroacetic acid, TFA). The remaining residue was dissolved in acetonitrile and purified by high-performance liquid chromatography on an Alliance 2695 module (Waters) with photodiode array detection on a Phenomenex Prodigy ODS3 (150 × 4.6 mm, 5 μm). The column was run with a linear gradient of 30–80% acidified methanol (0.05% TFA) over 15 min at 0.9 mL/min. In total, 6 peak fractions were collected, concentrated and tested in the TCL-BOA. A dominant bioactive peak fraction eluted at 6.957 min. This fraction was re-chromatographed on a LCQ Deca-XP LC-UV-MS system (Thermo Finnigan) using a Phenomenex Gemini C18 column (50 × 4.6 mm, 5 μm) and a linear gradient of acidified acetonitrile (0.05% TFA) ranging from 30 to 60% in 10 min. The peak representing the antibacterial compound **1** (Figure 4) was detected both, in positive electrospray ionization mode (ESI^+) and atmospheric pressure chemical ionization mode ($APCI^+$). Data were collected from *m/z* 100 to 220. The UV spectrum of **1** was in accordance with literature data for tropodithietic acid (TDA) [1]. The ESI^+-mass spectrum showed a molecular ion $[M + H]^+$ of 212.88 Da and a dominant fragment ion $[M + H - H_2O]^+$ at 194.89 Da. The $APCI^+$ mass spectrum showed a

dominant molecular ion $[M + H]^+$ of 212.94 Da and a dominant fragment ion $[M + H - CO2]^+$ at 169.15 Da. Collision induced MS^2 of the molecular ion in ESI^+ did not result in further fragmentation. These data were in direct accordance with the LC-MS data of TDA reported by Bruhn *et al.* [2].

For NMR analysis **1** was dissolved in DMSO-d6 (^{13}C) or benzene-d6 (^{1}H). Samples were analysed on a Bruker DPX 300 MHz system. The ^{13}C-NMR spectra in DMSO displayed eight carbon signals with one α, β-unsaturated enolic carbon, one carboxylic carbon, six aromatic carbons including two carbons connected with heteroatoms. The ^{1}H-NMR spectrum in benzene displayed three adjacent proton signals in a A,B,C spin pattern. Measured shifts of proton and carbon signals of **1** were in accordance with the data previously obtained for TDA [1].

References

1. Liang, I. Investigation of secondary metabolites of North Sea bacteria: Fermentation, isolation, structure elucidation and bioactivity. Ph.D. Thesis, University of Gottingen, Gottingen, Germany, 2003.
2. Bruhn, J.B.; Nielsen, K.F.; Hjelm, M.; Hansen, M.; Bresciani, J.; Schulz, S.; Gram, L. Ecology, inhibitory activity, and morphogenesis of a marine antagonistic bacterium belonging to the *Roseobacter* clade. *Appl. Environ. Microbiol.* **2005**, *71*, 7263–7270.

© 2011 by the authors; licensee MDPI, Basel, Switzerland. This article is an open access article distributed under the terms and conditions of the Creative Commons Attribution license (http://creativecommons.org/licenses/by/3.0/).
